# Identification of DNA Methyltransferase-1 Inhibitor for Breast Cancer Therapy through Computational Fragment-Based Drug Design

**DOI:** 10.3390/molecules26020375

**Published:** 2021-01-13

**Authors:** Ahmad Husein Alkaff, Mutiara Saragih, Shabrina Noor Imana, Mochammad Arfin Fardiansyah Nasution, Usman Sumo Friend Tambunan

**Affiliations:** Bioinformatics and Biomedicals Research Group, Department of Chemistry, Faculty of Mathematics and Natural Sciences, Universitas Indonesia, Depok 16424, West Java, Indonesia; ahmad.husein@sci.ui.ac.id (A.H.A.); mutiara.saragih@sci.ui.ac.id (M.S.); shabrina.noor@sci.ui.ac.id (S.N.I.); marfin.f@sci.ui.ac.id (M.A.F.N.)

**Keywords:** dengue, envelope protein, natural product, fragment growing, molecular docking simulation

## Abstract

Epimutation by DNA Methyltransferase 1 (DNMT1), an epigenetic regulator enzyme, may lead to the proliferation of breast cancer. In this report, 168,686 natural products from the PubChem database were screened and modified by in silico method to acquire the potential inhibitor of DNMT1. The initial screening of PubChem natural products using Lipinski’s and Veber’s rules of three and toxic properties have resulted in 2601 fragment candidates. Four fragments from pharmacophore-based molecular docking simulation were modified by utilizing FragFP and the Lipinski’s and Veber’s rules of five, and resulted in 51,200 ligands. The toxicological screening collected 13,563 ligands for a series of pharmacophore-based molecular docking simulations to sort out the modified ligands, which had the better binding activity and interactions to DNMT1 compared to the standards, SAH, SAM, and SFG. This step resulted in five ligand candidates, namely C-7756, C-5769, C-1723, C-2129, and C-2140. The ADME-Tox properties prediction showed that the selected ligands are generally better than standards in terms of druglikeness, GI absorption, and oral bioavailability. C-7756 exhibited a stronger affinity to DNMT1 as well as better ADME-Tox properties compared to the other ligands.

## 1. Introduction

Cancer is a chronic disease characterized by uncontrolled cell growth that can spread to tissues and other organs in the body [1]. Breast cancer is one type of cancer with many cases of death among female patients and continues to be a global medical issue. Even though the number of major medical advances have improved the treatment of primary breast cancer [2], it still contributes to 11.6% of the total cancer incidence burden worldwide, with approximately 2.1 million people suffering from breast cancer in 2018 [3]. In Indonesia, breast cancer continues to be the most common malignancy in women, with an incidence rate of 30.9% per total number of 188,231 new cases in 2018 [4].

**Figure 1:**
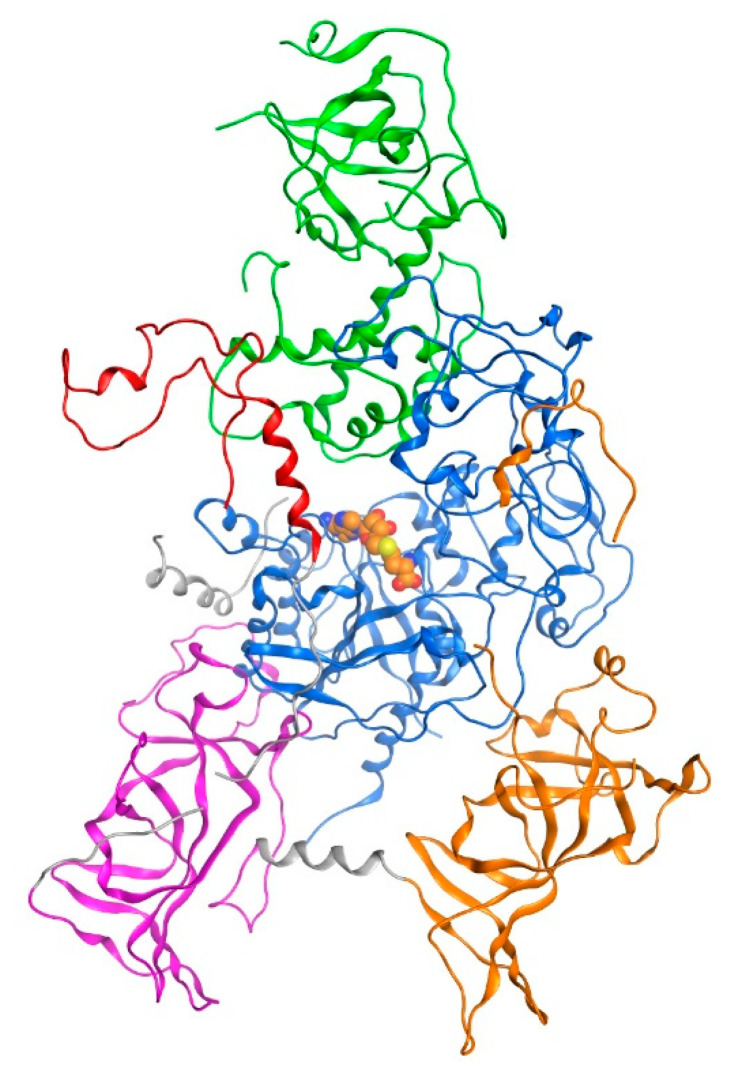
The 3D structure of the DNMT1 protein (PDB ID: 3AV5 [5]) with the RFTS, CXXC, Mtase, BAH1, and BAH2 domain, which are marked in green, red, blue, magenta, and orange, respectively. The SAH (orange-colored space-filling model) occupies a hydrophobic pocket in the MTase domain.

DNA Methyltransferase 1 (DNMT1) is an epigenetic regulator enzyme responsible for forming and maintaining DNA methylation patterns [5]. The epigenetic modification through methylation at cytosine residues of DNA plays essential role in regulating gene expression without altering the original DNA sequence [6]. The DNMT1 protein consists of five domains, which are: replication foci targeting sequence (RTFS) domain, CXXC zinc finger domain, bromo adjacent homology 1 (BAH1) domain, BAH2 domain, and MTase domains (Figure 1) [7]. During DNA methylation, the transfer of the adenosyl-l-methionine (SAM) methyl group (-CH3) to the 5′-cytosine position in the DNA sequence forming 5-methylcytosine (5mC) and S-adenosyl-l-homocysteine (SAH), occurs in the MTase domain [8]. 

In the normal cells, epigenetic regulation dictates the expression of oncogenes, which encourage the formation of cancer cells, and tumor suppressor genes that regulate the growth as well as the life cycle of a cell. Epimutation or epigenetic alteration is a change in the DNA methylation pattern occurring in various types of cancer where there is a massive upregulation in oncogene expression and a downregulation in tumor suppressor gene expression [9].

Epimutation is characterized by a decrease in the amount of global methylated DNA and an increase in DNA methylation in CpG (CG site) islands, a region containing a huge number of CpG dinucleotide repeats [10] or regions of DNA where a cytosine nucleotide is followed by a guanine in a linear sequence of bases along its 5′-3′ direction. CpG islands are DNA methylation regions in promoters known to regulate gene expression through transcriptional silencing of corresponding genes [11]. CpG islands usually extend for 300–3000 base pairs in mammalian genomes and are located within or close to approximately 50% of human promoters [12,13]. Generally, this area is not located in the gene promoter, and the CpG island is not usually methylated [14]. One of the leading causes of breast cancer is an increase in DNMT1 activity, which leads to the alteration in DNA methylation patterns and the increase in DNA methylation in CpG islands [15].

The natural product compound is known as a valuable source of medicines because of its bioactivity [16]. In addition to having an anticancer activity, the natural product compounds also pose an advantage as a drug due to their good bioavailability and therapeutic activity [16,17]. The natural product compounds such as epigallocatechin-3-gallate (EGCG), catechin, and quercetin exhibit a plausible activity as an inhibitor of DNMT1, resulting in DNA demethylation, which reactivates the tumor suppressor gene expression, thereby reducing the cancer cell growth rate [18,19]. Therefore, developing drug candidates as DNMT1 inhibitors is a potential strategy for breast cancer treatment.

The in silico method or computer-aided drug discovery and development is a rapidly developing field since it reduces the cost and development time in drug development [20]. The molecular docking and dynamic simulations have been regularly utilized and developed to analyze interactions, affinity, and stability of a ligand targeting other biomolecules [21]. On the other hand, the in silico method also makes the characterization of absorption, distribution, metabolism, excretion, and dan toxicity (ADMET) of studied compounds possible [22]. 

## 2. Materials and Methods

**Figure 2:**
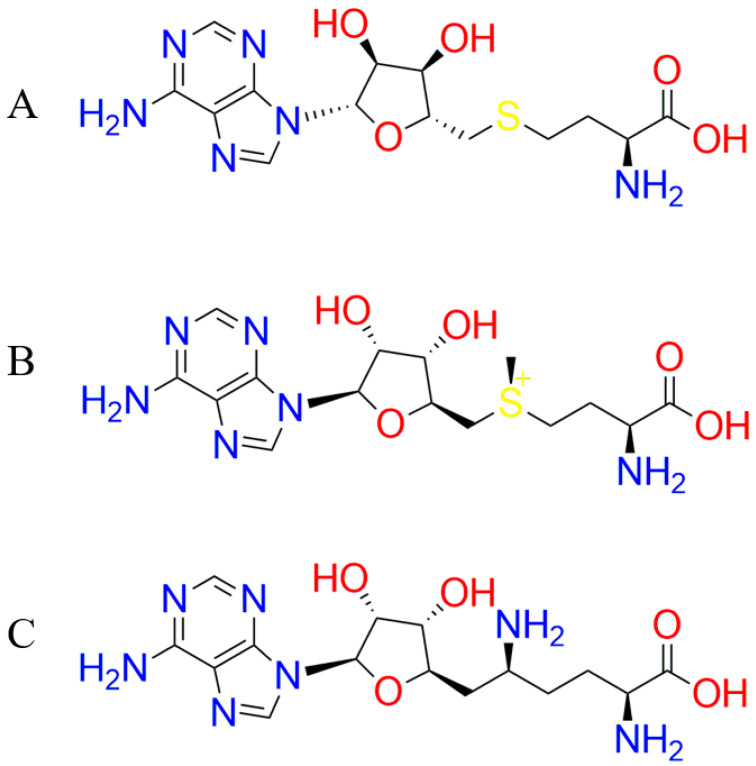
The 2D structure of S-Adenosyl-l-homocysteine—SAH (**A**), S-Adenosyl methionine—SAM (**B**), and Sinefungin—SFG (**C**).

Four protein structures of DNMT1 from RCSB PDB (https://www.rcsb.org/) with PDB ID: 3AV5 [23], 3AV6 [23], 3PTA [24], 4WXX [7], and two modeled DNMT1 structures from GenBank (https://www.ncbi.nlm.nih.gov/genbank/) (Accession number: AAH92517.1) [25] were used as the protein targets. Natural product compounds from PubChem database (https://pubchem.ncbi.nlm.nih.gov/): alkaloids (39,887), flavonoids (20,393), phenolics (46,268), saponins (5077), tannins (1042), and terpenes (55,979) were retrieved as the drug lead. The standard ligands, namely SAH, SAM, and sinefungin (SFG) with the ZINC15 ID of ZINC000001532516, ZINC000004214738, and ZINC000004217451, respectively, were obtained from the ZINC15 database (Figure 2) [26]. In this research, some software, such as Swiss-Model (https://swissmodel.expasy.org/), MOE 2014.09 [27], DataWarrior 4.5.2 [28], OSIRIS Property Explorer (https://www.organic-chemistry.org/prog/peo/) [29], Toxtree 2.6.6 [30], AdmetSAR (https://lmmd.ecust.edu.cn/admetsar1), and SwissADME (http://swissadme.ch/), were utilized to conduct the theoretical calculation. 

### 2.1. Pre-Docking Preparation

The 3D structure of DNMT1 proteins was prepared by using MOE 2014.09. Small molecules such as zinc and sulfate ions from protein targets were removed. Then, the potential setup was arranged with Amber10: EHT forcefield and R-field solvation. The “LigX” protocol was performed with a tether strength of 100,000, an RMS gradient value of 0.05 kcal/molÅ, and the unchecked “Allow ASN/GLN/HIS ‘Flips’ in Protonate 3D” option while the rest parameters were set in default.

The natural product compounds from the PubChem database were chosen as a fragment library. The fragment library was also subjected to the initial toxicological screening and the structure optimization using DataWarrior 4.5.2 and MOE 2014.09, respectively. All fragments were subjected to initial pharmacological screening of toxicity and a Rule of 3 (RO3) filter [31]. Structure optimization took place by arranging the potential setup to the MMFF94x force field with R-field solvation. Then, by using the selected “Presence Existing Chirality” and RMS gradient 0.001 kcal/mol/Å^2^ parameters, the fragment library was subjected to the default “Wash” and “Energy Minimization” process. The standard ligands, namely SAM, SAH, and SNF, were also subjected to the same structure optimization process.

The last step in pre-docking preparation was to determine the pharmacophore mapping of DNMT1. It was created using the standard procedure of the Protein-Ligand Interaction Fingerprints (PLIF) method in MOE 2014.09 software. Seven DNMT1 protein structures with their respective ligands in the binding sites were superposed. Protein–ligand interactions were mapped to find the conserved interaction, which served as the pharmacophore feature. As a result, the pharmacophore query was established at the end of the process.

### 2.2. Molecular Docking Simulation of the Fragment

The screened fragments were subjected to two-steps pharmacophore-based rigid molecular docking simulation against DNMT1 protein using MOE 2014.09 software. The “Rigid Receptor” protocol with pharmacophore pose prediction was utilized with the former simulation using 30 repetitions, while the latter used 100 repetitions. In both simulations, the rescoring of simulated poses was done using a London dG scoring function followed by a pose refinement by Force field algorithm along with GBVI WSA dG rescoring function in which the root-mean-square deviation (RMSD) was calculated. Every fragment with an RMSD value lower than 2.0 Å, attached inside the binding site, and forming significant H-bonds was chosen as the fragment candidates.

### 2.3. Fragment Growing

In this research, the DataWarrior 4.5.2 software with the fragFP and the Lipinski’s and Veber’s rules of fives (molecular weight ≤ 500 Da, −0.5 ≤ Log P ≤ 5.6, H acceptor ≤ 10, H donor ≤ 5, TPSA ≤ 140 Å^2^, rotatable bond count ≤ 10) were used as the parameters to generate ligands from the previously screened fragments. Afterwards, any ligands deemed to be identified as potentially mutagenic, tumorigenic, irritant, or harmful for the reproductive system and had a druglikeness score below 0 were omitted from further analysis. 

### 2.4. Molecular Docking Simulation of the Ligand

The screened ligands were subjected to the same structure optimization as the fragment library. Then, the series of molecular docking simulations by using MOE 2014.09 software of the screened ligands and standard compounds followed similar steps to the fragment library. Then, the series of molecular docking simulations using “Virtual Screening”, “Rigid Docking”, and “Induced Fit” protocols were carried out sequentially along with pharmacophore pose prediction, London dG scoring function, and the retain value of 1, 30, and 100 repetitions, respectively. Forcefield AMBER 10: EHT algorithm and GBVI WSA dG rescoring functions were selected as the parameter for pose refinement and rescoring processes. The standard ligands were also subjected to the same simulation to compare the docking protocol employed to predict the binding orientation of the SAH, SAM, and SFG in the DNMT1 binding pocket. In each simulation, all the ligands were ranked based on ∆Gbinding energy and RMSD values. Ligands with a ∆Gbinding energy higher than standard ligands and RMSD higher than 2.0 Å were excluded.

### 2.5. ADME-Tox Analysis

The molecular properties, druglikeness, and drug score of the standard compounds and ligands were observed using DataWarrior and OSIRIS property explorer [28,29]. Furthermore, its Absorption, Distribution, Metabolism, Excretion, and Toxicity (ADME-Tox) profile was computationally predicted using Toxtree v2.6.6, AdmetSAR, and SwissADME software [32,33,34].

## 3. Results

### 3.1. Initial Toxicological Screening

About 168,686 natural product compounds were retrieved from the PubChem database. The parameters used to filter the compounds in this step are the Lipinski’s and Veber’s rules of three (molecular weight ≤ 200 Da, −0.5 ≤ Log P ≤ 3, H acceptor ≤ 3, H donor ≤ 3, TPSA ≤ 60 Å^2^, rotatable bond count ≤ 3), druglikeness > 0, and toxic properties (have no mutagenic, tumorigenic, irritant, and damaging reproductive system). Only 2601 compounds passed the filters of this analysis (Table 1).

### 3.2. Molecular Docking Simulation of the Fragment

The structure of DNMT1 (351–1600) with PDB ID 3AV5 was determined using X-Ray diffraction with a resolution of 2.66 Å [28]. The SAH was attached to the DNMT1 binding pocket located in the MTase region, which comprised of 28 residues, namely Asp1146, Val1147, Phe1148, Ser1149, Gly1150, Cys1151, Gly1152, Gly1153, Leu1154, Ser1155, Ile1170, Glu1171, Met1172, Trp1173, Ala1176, Glu1192, Asp1193, Cys1194, Asn1195, Gly1225, Gly1226, Pro1228, Leu1250, Glu1269, Arg1576, Asn1580, Ala1581, Val1582. According to the obtained PDB structure, SAH was observed to form ten hydrogen bond interactions with Asp1146, Phe1148, Gly1153, Leu1154, Glu1171, Asp1193, Cys1194, Asn1580, and Val1582: two of which were with Glu1171. Identifying the native substrate and its binding site is an essential step in designing an effective inhibitor against a particular target protein.

**Figure 3:**
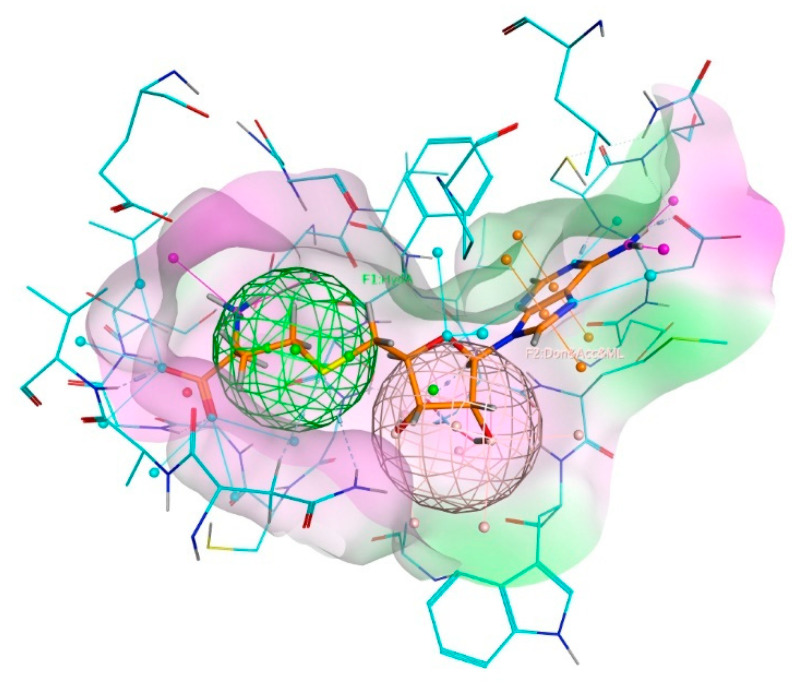
The pharmacophore model of MTase binding pocket of DNMT1 constructed from SAH as the reference compound. The HydA (green) and Don&Acc&ML (pale pink) marked the designated hydrophobic and H-bond donor, H-bond acceptor, and metal ligator annotation, respectively. The purple and green surface areas illustrate the hydrophilic and hydrophobic regions, respectively.

The pharmacophore query was generated from six DNMT1 protein structures, namely 3AV5, 3AV6, 3PTA, 4WXX, AHH92517.1a, and AHH92517.1b along with SAH, SAM, and SFG as the reference compounds of protein–ligand interactions. The PLIF protocol generated three queries: HydA, Don&Acc&ML, and Acc&ML. Then, the reference molecules were reattached to the query by utilizing the “Pharmacophore Searching” feature on MOE 2014.09 to validate the generated pharmacophore. Only the combination of HydA and Don&Acc&ML was able to detect SAH, SAM, and SFG. Therefore, this query was chosen as the pharmacophore model for the molecular docking simulation (Figure 3).

**Figure 4:**
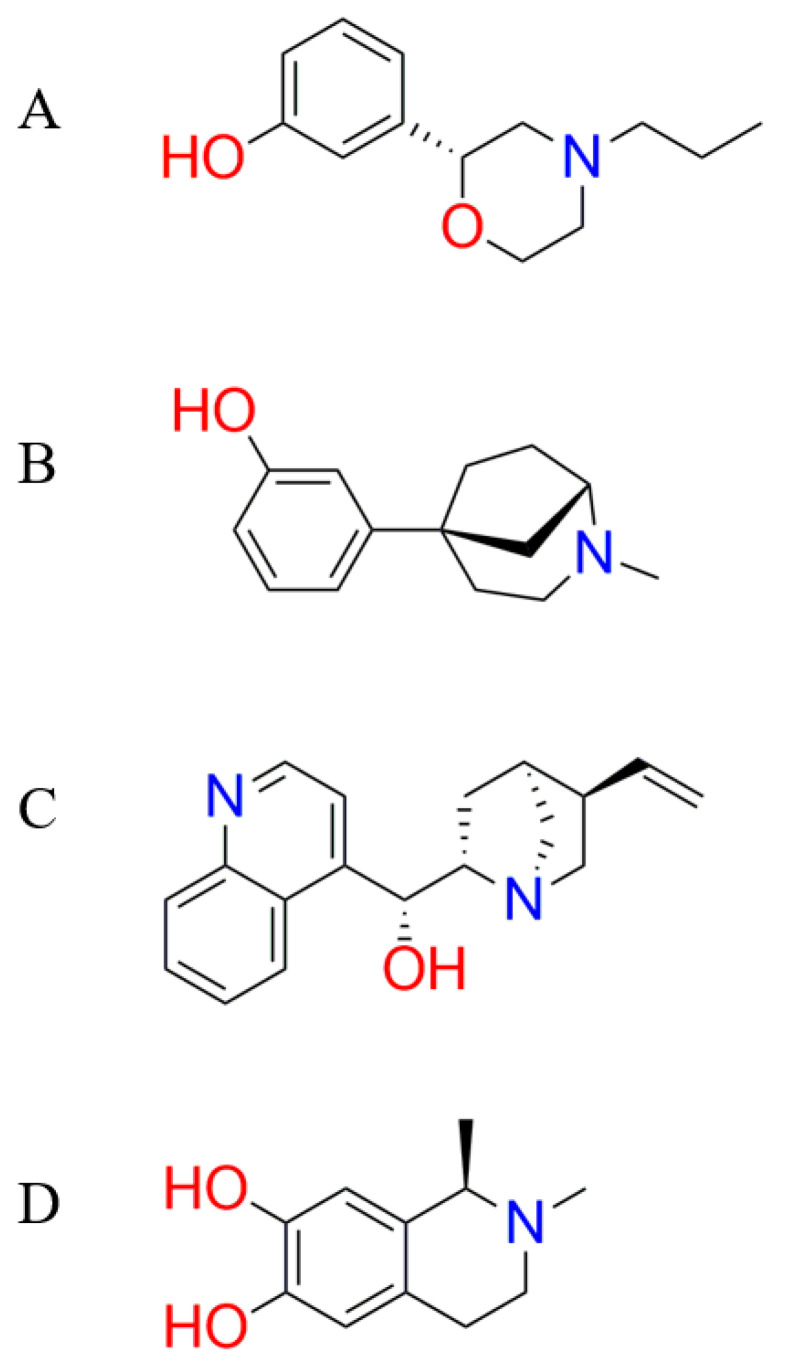
The structure of the selected fragments for fragment growing: 3-[(2*R*)-4-propyl-2-morpholinyl]phenol (**A**), 3-(2-methyl-2-azabicyclo[3.2.1]oct-5-yl)phenol (**B**), α-quinidine (**C**), and (*R*)-*N*-methylsalsolinol (**D**).

The rigid docking, which simulates the “lock and key” interaction of protein and ligand, was used as the protocol for a two-step molecular docking simulation to identify a potential fragment for further modification. From each molecular docking simulation, the 2.0 Å cut off for RMSD value was selected because the docking results with RMSD > 2.0 Å were identified to be less reproducible in subsequent analyses [35]. The RMSD is as measure of similarity between the real ligand position in the receptor and the computed position of the docking ligand [36]. In molecular docking simulations, the RMSD value is defined to compare the docked conformation with the reference conformation or with other docked conformations [37]. A ligand-receptor molecular docking simulation with an RMSD value below 2 Å is considered as a conformation with a high docking accuracy [36]. The simulation was performed with the retain value of 30 and 100 repetitions, respectively. The former simulation eliminated 2146 out of 2601 fragments, while the latter retained 287 out of 455 fragments. From 287 fragments which passed the molecular docking simulations, four fragments were chosen, namely 3-[(2*R*)-4-propyl-2-morpholinyl]phenol, 3-(2-methyl-2-azabicyclo[3.2.1]oct-5-yl)phenol, α-quinidine, and (*R*)-*N*-methylsalsolinol (Figure 4). These fragments were chosen because they attached to the protein binding site following the feature of the pharmacophore query and formed the highest number of hydrogen bonds compared to the other fragments.

### 3.3. Fragment Growing

Fragment growing is the process of constructing a reasonable molecular structure around a fragment. It typically starts with a single fragment and proceeds by expanding the molecular structure to probe further parts of the protein binding site in order to increase its affinity [38]. The fragment growing method from the DataWarrior software was able to generate 12,800 ligands from each selected fragment, totaling about 51,200 ligands. However, only 13,563 ligands were cleared for the next phase analysis after the druglikeness and toxic property screening (Table 2).

### 3.4. Molecular Docking Simulation of the Ligand

**Figure 5:**
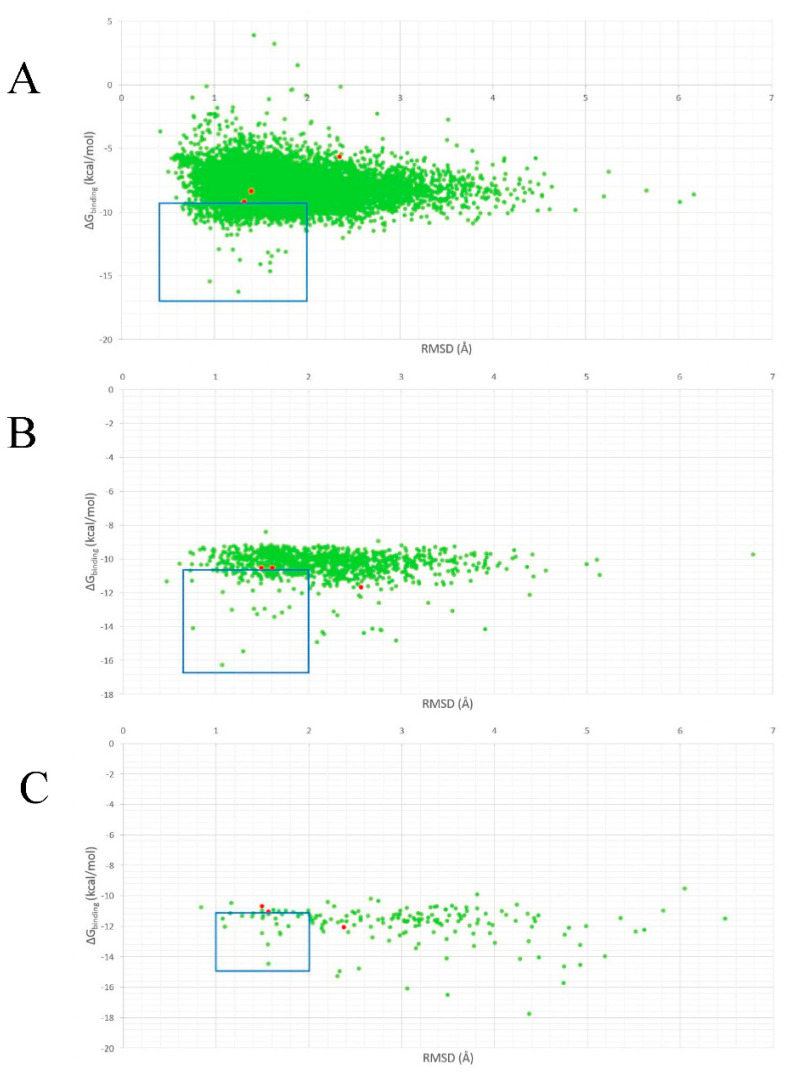
Distribution of ΔGbinding energy and RMSD value distribution of ligand and standard compounds during virtual screening (**A**), rigid (**B**), and flexible (**C**) docking simulations. The ligands are marked in a green dot while the standards are marked with red dots. About 1133, 135, and 22 ligands, which had a ΔGbinding energy lower than standards and an RMSD value lower than 2.0 Å, passed the respective virtual screening, rigid, and flexible docking simulations (blue box).

The 13,563 ligands and three standards underwent three rounds of molecular docking simulation against the DNMT1 MTase binding pocket using MOE 2014.09 software. A “Virtual Screening” protocol was used as the first protocol in the sequence of docking simulation to instantaneously identify ligands that can fit into the binding pocket and its determined pharmacophore. The second docking simulation utilizes a “Rigid Receptor” protocol where the ligands can move freely in the rigid binding pocket to find the optimal binding pose. In the last docking simulation, both the protein binding pockets and the ligands were moved flexibly in a simultaneous manner to probe for the optimum protein-ligand conformation through the “Induced Fit” protocol. In each step, ligands, which had RMSD value lower than 2.0 and a Gibbs binding (∆Gbinding) energy lower than the standards, passed the respective simulations (Figure 5). The ∆Gbinding energy was also used as a parameter in addition to the RMSD value because it represented ligand affinity to the target protein and the spontaneity of the protein–ligand complex formation. Thus, in this research, ligands with low ∆Gbinding energy were said to have a better affinity and reacted more spontaneously to DNTM1.

**Figure 6:**
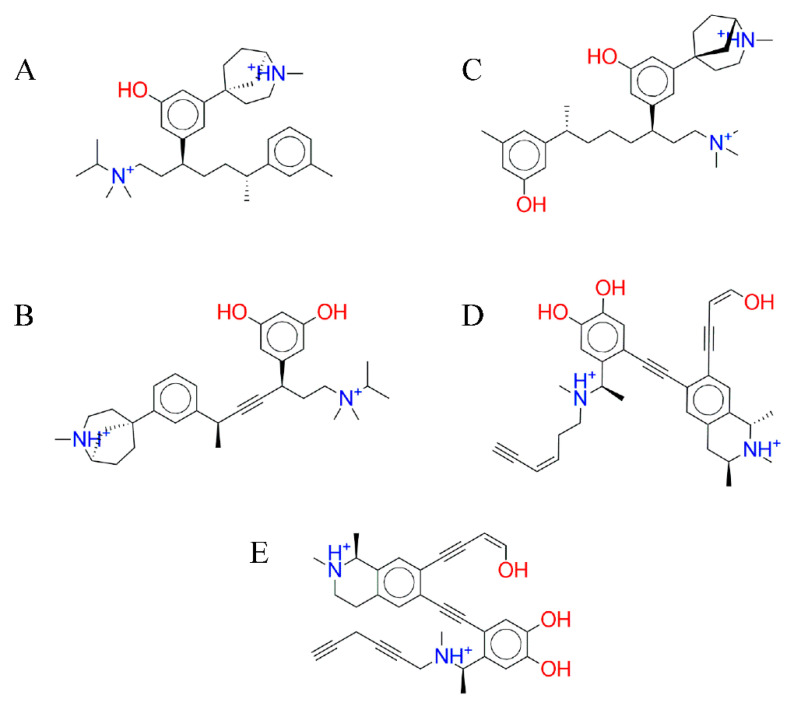
The structure of five ligands from the series of molecular docking simulations: C-7756 (**A**), C-5769 (**B**), C-1723 (**C**), C-2129 (**D**), and C-2140 (**E**). C-7756, C-5769, and C-1723 are derived from 3-(2-methyl-2-azabicyclo[3.2.1]oct-5-yl)phenol, while C-2129 and C-2140 are derived from (*R*)-*N*-methylsalsolinol.

At the end of the simulation, 22 ligands had successfully passed three rounds the molecular docking simulations. These ligands were ranked based on their ΔGbinding energy. Then, five top ligands, namely C-7756, C-5769, C-1723, C-2129, and C-2140, were considered to have a high potency as a DNMT1 MTase inhibitor (Table 3). C-7756, C-5769, and C-1723 were grown from 3-(2-methyl-2-azabicyclo[3.2.1]oct-5-yl)phenol, while C-2129 and C-2140 were grown from (*R*)-*N*-methylsalsolinol. Figure 6 represent the structure of five potential ligands generated from this research.

**Figure 7:**
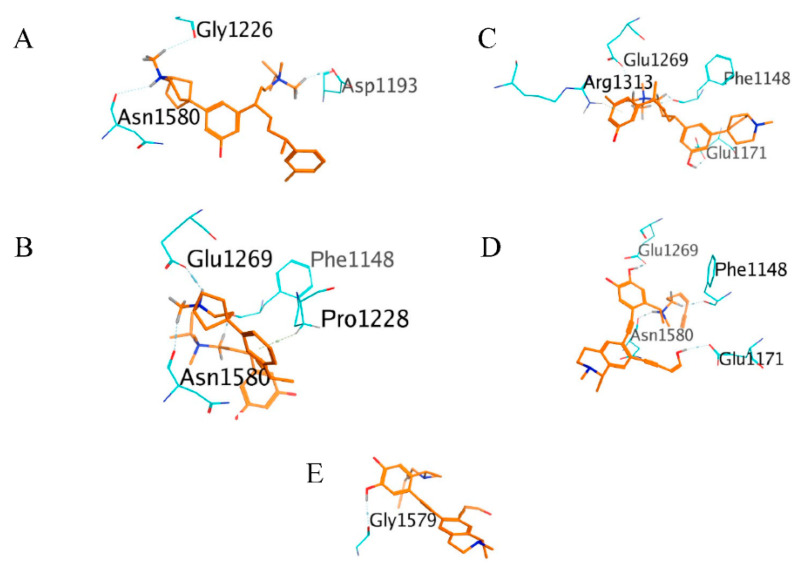
The 3D interaction of the best ligand from a flexible docking simulation marked in orange: C-7756 (**A**), C-5769 (**B**), C-1723 (**C**), C-2129 (**D**), and C-2140 (**E**). The amino acid residues of DNMT1, which formed hydrogen and H-π bonds, are presented in light blue color.

C-7756, which has an IUPAC name of (1*R*,5*S*)-5-(3-hydroxy-5-((3*S*,6*R*)-1-(isopropyldimethylammonio)-6-(m-tolyl)heptan-3-yl)phenyl)-2-methyl-2-azabicyclo[3.2.1]octan-2-ium, interacted with 16 amino acid residues, three of which were hydrogen bonds with Asp1193, Gly1226, and Asn1580 of the DNMT1 MTase binding site (Figure 7A).

C-5769, which has an IUPAC name of (1*R*,5*S*)-5-(3-((2*S*,5*R*)-5-(3,5-dihydroxyphenyl)-7-(isopropyldimethylammonio)hept-3-yn-2-yl)phenyl)-2-methyl-2-azabicyclo[3.2.1]octan-2-ium, interacted with 25 amino acid residues, three of which were hydrogen bonds with Phe1148, Glu1269, and Asn1580, and one of which was an H-π bond with Pro1228 of the DNMT1 MTase binding site (Figure 7B).

C-1723, which has a IUPAC name of (1*S*,5*R*)-5-(3-hydroxy-5-((3*R*,7*R*)-7-(3-hydroxy-5-methylphenyl)-1-(trimethylammonio)octan-3-yl)phenyl)-2-methyl-2-azabicyclo[3.2.1]octan-2-ium, interacted with 25 amino acid residues, two of which were hydrogen bonds with Glu1171 and Glu1269, the other two of which were hydrogen bonds with Phe1148 and one of which was an H-π bond with Arg1313 of the DNMT1 MTase binding site (Figure 7C).

C-2129, which has a IUPAC name of (1*S*)-6-((2-((1*R*)-1-(((*Z*)-hex-3-en-5-yn-1-yl)(methyl)ammonio)ethyl)-4,5-dihydroxyphenyl)ethynyl)-7-((*Z*)-4-hydroxybut-3-en-1-yn-1-yl)-1,2-dimethyl-1,2,3,4-tetrahydroisoquinolin-2-ium, interacted with 25 amino acid residues, four of which were hydrogen bonds with Phe1148, Glu1171, Glu1269, and Asn1580 of the DNMT1 MTase binding site (Figure 7D).

C-2140, which has a IUPAC name of (1*S*)-6-((2-((1*R*)-1-(hexa-2,5-diyn-1-yl(methyl)ammonio)ethyl)-4,5-dihydroxyphenyl)ethynyl)-7-((*Z*)-4-hydroxybut-3-en-1-yn-1-yl)-1,2-dimethyl-1,2,3,4-tetrahydroisoquinolin-2-ium, interacted with 25 amino acid residues, one of which as a hydrogen bond with Gly1579 of the DNMT1 MTase binding site (Figure 7E). 

**Figure 8:**
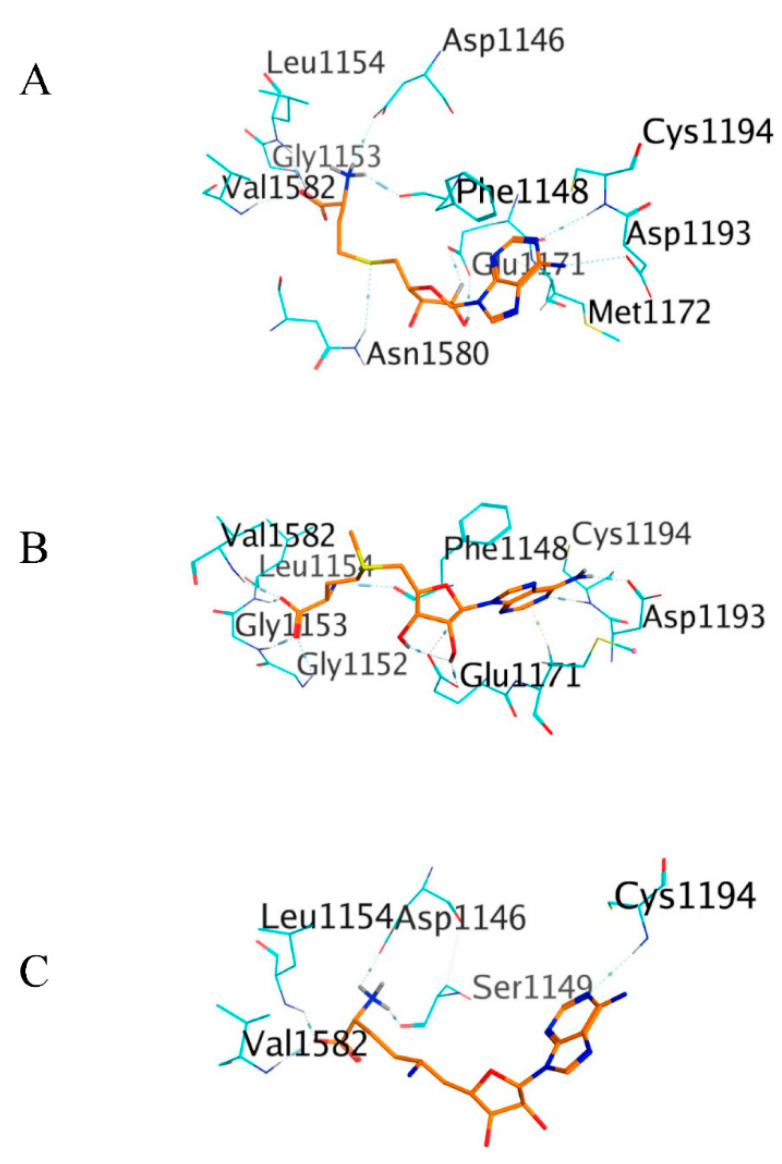
The 3D interaction of standard compounds from flexible docking simulation marked in orange: SAH (**A**), SAM (**B**), and SFG (**C**). The amino acid residues of DNMT1, which formed hydrogen and H-π bonds, are presented in light blue color.

In terms of the binding pose, C-1723 has been shown to fit well inside the DNMT1 Mtase binding pocket in a similar conformation to the standard compounds (Figure 8). The (*R*)-7-(3-hydroxy-5-methylphenyl)-*N*,*N*,*N*-trimethyloctan-1-aminium functional group of C-1723 buried deep in the polar region of DNMT1 MTase binding pocket and formed three hydrogen bonds and one H-π bond. Its phenolic functional group acted as an anchor by forming a hydrogen bond with Glu1171 at the center of the binding pocket. Meanwhile, the (1*S*,5*S*)-2-methyl-2-azabicyclo[3.2.1]octan-2-ium functional group of C-1723 was attached in the nonpolar region of the MTase binding pocket.

### 3.5. ADME-Tox Analysis

In this research, the molecular properties of the selected ligands from previous docking simulation results were carried out by using DataWarrior software and OSIRIS Property Explorer online web service. These softwares not only predicted the molecular properties, but also the assume drug scores, which translated as an ability of compounds to become a drug, based on their molecular properties and druglikeness value. The result of these tests showed that all five ligands did not violate any Lipinski’s RO5, while all standard ligands have a logP value lower than −0.5, a hydrogen bond acceptor more than 10, and a TPSA higher than 140 Å^2^. Interestingly, Compound C-7756 was the only compound in this test that has a positive druglikeness value, sitting in 1.45, while others have a negative druglikeness value, with SAM, SAH, and SFG at a negative druglikeness value among all with −9.09, −18.44, and 19.10, respectively. The positive result of Compound C-7756 might due to its low TPSA compared to the other four NP ligands (23.47 Å^2^, compared to 43.70 Å^2^ for both Compound C-5769 and Compound C-1723, and 67.17 Å2 for both Compound C-2129 and C-2140). Hence, Compound C-7756 has the highest drug score among all five of the best NP ligands at 0.41, despite still being lower than both SAH and SFG at 0.42. These results can be seen in Table 4.

The mutagenicity and carcinogenicity potency of the selected ligands were identified using Toxtree v2.6.6 software. This software predicts these properties based on the chemical structures that the ligand possessed, which were then compared to the carcinogenic/mutagenic database that corresponded to the software. Toxtree v2.6.6 analyzes the ligand carcinogenicity based on three different mechanisms: genotoxic, non-genotoxic, and quantitative structure–activity relationship (QSAR) carcinogenicities [32,39]. Meanwhile, the ligand mutagenicity was predicted based on the Ames test, which includes the usage of Salmonella typhimurium as the original sample [40]. According to the result shown in Table 5, all five of the best ligands did not show any mutagenic nor carcinogenic properties, as they did not possess any fragments that may lead to carcinogenicity or mutagenicity. In contrast, all standard ligands, SAH, SAM, and SFG, were predicted to become the genotoxic carcinogenic agents, which may happen due to primary aromatic amines that these ligands possessed in their respective molecular structures.

According to the results in Table 6, all five ligands were predicted to act as a substrate of P-gp, while they also did not possess any inhibitor potency of P-gp as well. Moreover, these ligands acted as a CYP450 substrate, particularly as a CYP3A4 substrate. These results were forecasted in the beginning since the P-gp substrate is much more likely to behave as a CYP3A4 substrate as well [41]. In addition, the biodegradable potencies of all five ligands were also identified as well, since the non-biodegradable compounds should be cautiously monitored since they may harm the environments, especially posing a risk to aquatic life such as fish [42]. In this study, however, all ligands, including the standard ligands, have no biodegradability capacity over the biological organism. Finally, the AMES toxicity and the carcinogenicity predictions of these ligands were also observed, and were predicted as non-AMES toxicant and non-carcinogenic agents. These results were similar to those from the previous test obtained from Toxtree v2.6.6 software.

Finally, the oral bioavailability, PAINS, and synthetic accessibility predictions were predicted in this study as well. These predictions were performed using the SwissADME web service [34]. The first indicator of this prediction was the gastrointestinal absorption, which was influenced by the substance physiochemical state [43]. Molecular traits such as MW, logP, and TPSA profoundly affected the capability of GI absorption in the human body, which inspired the Lipinski’s RO5, as well as Veber’s and Egan’s rule to be applied in determining the potential substance that can be absorbed well in an oral administration system [44,45,46]. In this study, the SwissADME prediction demonstrated that all five ligands have a high GI absorption towards the human body, and it was later confirmed that all ligands also passed the Veber’s, Egan’s, and Lipinski’s RO5 as well, violating none of these rules according to this result. Contrariwise, neither SAH, SAM, nor SFG have a high GI absorption, probably due to their high TPSA and low logP, which ultimately violate those rules and decrease their GI absorption. However, despite these results, all ligands were shown to have a moderate bioavailability score at 0.55.

The final two predictions of SwissADME on five best and three standard ligands were focused on the pan-assay interference (PAINS) and synthetic accessibility (SA) predictions, and according to the results, Compound C-2129 and Compound C-2140 have a positive result on the PAINS assay, mainly due to catechol fragments that reside in both compounds. These results may lead to false-positive results in the high-throughput screen (HTS) for biological targets [47]. Thus, these compounds should be noted with care when they are going to be screened through the HTS method. However, compared to the other three ligands, both Compound C-2129 and Compound C-2140 have lower SA values, which means that these ligands were easier to be synthesized compared to others, consequently reducing the cost and time to make these compounds in the laboratory. All results from SwissADME software can be seen in Table 7.

## 4. Discussion

Molecular profiling analysis was conducted to differentiate the subtype of breast cancer, namely normal breast-like, basal-like, luminal A, luminal B, and HER-2 breast cancer [48]. Drug discovery has been explored from the previous research to treat breast cancer patients. For example, Pertuzumab and Trastuzumab are two examples of drugs used in HER2-positive breast cancer patients [49]. DNMT1 is most highly expressed in basal-like breast cancer. It is also distinctly expressed in other types of breast cancer according to their molecular and stromal subtypes [50].

DNMT1 is an epigenetic regulator enzyme responsible for forming and maintaining DNA methylation patterns. In mammalian, DNMT1 is an essential enzyme in the mammalian genome functional system. Studies of DNA methylation can provide information in the current biomedical sciences, such as carcinogenesis, host infection by different viruses, cell differentiation, autoimmune diseases, different types of mental illness, neurological disorders, and environmental toxicology [51,52]. Hypermethylated promoters due to extensive DNA methylation may serve as a biomarker. Unlike the other irreversible genetic alterations, DNA methylation is reversible, making it a compelling approach for breast cancer therapy [53].

The DNMT inhibitors can provide novel and efficacious solutions for patients who suffer from hematological malignancies but also other cancer types. Two azanucleosides-based DNMT inhibitors have been approved by the US Food and Drug Administration (FDA) in 2013, namely decitabine (5 aza 2′ deoxycytidine) and azacytidine (Vidaza; Celgene). At lower doses, decitabine and azacytidine induce a strong demethylating effect, leading to re-expression of aberrantly silenced genes associated with reduced proliferation, apoptosis, senescence, and cell differentiation. Despite their clinical efficacy, DNA damage is observed after the incorporation of higher doses of decitabine and azacytidine. Moreover, their limitations extended to high toxicity, instability in physiological media, and poor bioavailability [54].

Natural products have been extensively studied for their function demethylating agents or DNMT inhibitors. Several flavonoids, anthraquinones, polyphenols, and other natural products have been known to inhibit the DNA methylation process by DNMTs, thus decreasing the silencing of various genes involved in tumorigenesis. This may lead to the re-expression of oncogenes in diverse cancer cell lines. Laccaic acid A and epigallocatechin-3-gallate have demonstrated their potent activity as DNMT1 competitive inhibitors with submicromolar IC50 values [18,19]. Despite the current advancement in the development of DNMT1 inhibitors, the pursuit to discover peculiar compounds targeting DNMTs, which are not only effective but are also more selective and less toxic, should be continued. Meanwhile, designing an inhibitor for which action relies on the reactivation of abnormally silenced tumor suppressor genes would be quite challenging. Hence, targeting DNMTs is a more feasible approach.

Fragment growing is an approach to improving potency and pharmacological properties by the addition of functional groups or substituents to the fragmented core. It is used to optimize their structure into favorable interactions with the binding site residues. The natural product compounds were chosen as a fragment library. All fragments were subjected to initial pharmacological screening with toxicity and Rule of 3 (RO3) filter. The “Rule of three” states that fragments should have a molecular weight ≤ 300 Da, cLogP ≤ 3, a hydrogen bond acceptor count ≤ 3, and number of hydrogen bond donors ≤ 3. Their analysis also indicates that using additional filters, such as rotatable bonds count ≤ 3 and the total polar surface area (TPSA) ≤ 60 Å, would give more desirable fragment-like compounds [31,55,56].

After screened natural product compound using DataWarrior and also MOE software, α-quinidine, 3-[(2*R*)-4-propyl-2-morpholinyl]phenol (*R*)-*N*-Methylsalsolinol and 3-(2-methyl-2-azabicyclo[3.2.1]oct-5-yl) phenol were selected after initial toxicological screening and molecular docking simulations. These four compounds are part alkaloid when (*R*)-*N*-Methylsalsolinol is a member of isoquinoline, a heterocyclic alkaloid. (*R*)-*N*-Methylsalsolinol, a dopamine-derived neurotoxin selective to dopamine neurons, is known to induce parkinsonism in rats. These four fragment structures were generated using a fragment growing method and were screened using the same software, DataWarrior. After ligands from fragment growing were collected, the docking simulations (Virtual screening, rigid docking, and flexible docking) between protein and ligand were initiated. Based on Table 3, we could observe the five best ligands based on their dG binding and RMSD. C-7756, C-5769, and C-1723 are derived from 3-(2-methyl-2-azabicyclo[3.2.1]oct-5-yl)phenol. C-2129 and C-2140 are derived from (*R*)-*N*-Methylsalsolinol. 

The absorption, distribution, metabolism, excretion, and toxicity (ADME-Tox) property predictions of any drug candidates become an inevitable method in drug discovery and development (CADDD). It is estimated that 30% of drug attrition has been caused by drug failures, mainly triggered by the unwanted ADME-Tox properties of the drug itself [57]. However, neither in vitro nor in vivo investigations to determine these properties are inexpensive and time effective. This is because they heavily contribute to the high cost of developing new drugs nowadays, which approximately takes about 2.6 billion USD and 14–20 years from the initial phase in the laboratory until it is widely marketed [20,58]. In recent years, computational-based ADME-Tox predictions, which offer a valuable, safe, cheap, and rapid method to accurately determine the molecule properties based on the structural alerts, for instance, had become an invaluable tool in CADD and have been routinely performed before the drug candidates had been synthesized in a wet laboratory [59,60]. In this study, some software has been carried out to identify the ADME-Tox properties of the selected ligands from docking simulations results, such as DataWarrior [28,29], Toxtree v2.6.6 [32], admetSAR [33], and SwissADME [34].

The molecular properties of the ligands may determine their ability to be easily absorbed into the human body via oral administration. Hence, Lipinski’s Rule of Five (RO5) was popularized and has possibly been the most leading, yet simple concept in CADDD and medicinal chemistry fields in the last few decades [56,61]. This rule revolves around five different molecular properties; logP, molecular weight (MW), hydrogen bond acceptor and donor, and topological polar surface area (TPSA). Overall, Lipinski’s RO5 stated that any compound has a high probability of having poor permeability and absorption through oral administration when the compound has either a molecular weight higher than 500 Dalton, a logP higher than 5.0, a hydrogen bond acceptor more than 10, or a hydrogen bond donor more than 5 [45,56]. Additionally, a higher TPSA value than 140 Å^2^ is also accountable for low oral absorption of the drug molecule as well [46].

The potency of a compound to become either a substrate or an inhibitor for both P-glycoprotein (P-gp) and Cytochrome (CYP) 450 enzymes also determines effectiveness and efficiency when it acts like a drug in the human body. P-gp is a drug transporter that plays an imperative role in preventing toxic substances by limiting its absorption when administered orally; this protein also plays a significant part in drug–drug interaction [62]. Furthermore, any compound that can work as a P-gp substrate may affect its functions, either as an inducer or an inhibitor, which can decrease and increase its bioavailability in the human body, respectively [41]. On the other hand, the CYP450 enzymes are one of the essential metabolizing enzymes that are mainly involved in various oxidizing reactions for xenobiotic compounds [63]. Out of five common CYPs involved in these reactions, the CYP450 3A4 is the most important one, and is accountable in the metabolizing processes for more than half the marketed drugs in the world [64]. In this study, these properties can be identified using admetSAR web services [33].

## 5. Conclusions

Our results showed that C-7756, C-5769, C-1723, C-2129, and C-2140 have a higher affinity to DNMT1 compared to the standards (SAH, SAM, and SFG), which is determined by their lower ∆Gbinding. Moreover, the selected ligands have pharmacological advantages in terms of druglikeness, GI absorption, and oral bioavailability compared to the standards. Having the lowest ∆Gbinding and least-unwanted ADME-Tox properties, our results indicated that C-7756 has the potential to be a drug lead for inhibiting DNMT1 for breast cancer therapy. Finally, our results must be further examined through molecular dynamic simulation as well as through in vitro and in vivo methods to investigate its potential in the biological condition.

## Figures and Tables

**Table 1:** Natural products compound prepared as a fragment database. DataWarrior software was utilized to assess the parameters for the initial pharmacological filters, which includes the Lipinski’s and Veber’s rules of three (molecular weight ≤ 300 Da, −0. 5 ≤ Log P ≤ 3, H acceptor ≤ 3, H donor ≤ 3, TPSA ≤ 60 Å^2^, rotatable bond count ≤ 3), druglikeness > 0, and toxic properties (no mutagenic, tumorigenic, irritant, and reproductive effect).

Compound Classification	Natural Product Compounds Collected from the PubChem Database	Natural Product Compounds which Passed the Initial Pharmacological Filters
Alkaloids	39,887	1949
Flavonoids	20,393	191
Phenolics	46,268	138
Saponins	5077	2
Tannins	1042	0
Terpenes	55,979	321

**Table 2:** Ligands generated from fragment growing method using DataWarrior software. The same software was also used to perform toxicological screening, which includes druglikeness > 0, and toxic properties (no mutagenic, tumorigenic, irritant, and reproductive effect).

Fragment	Number of Ligands Generated from DataWarrior Software	Ligands which Passed the Toxicological Screening
3-[(2*R*)-4-propyl-2-morpholinyl]phenol	12,800	163
3-(2-methyl-2-azabicyclo[3.2.1]oct-5-yl)phenol	12,800	8041
α-quinidine	12,800	3143
(*R*)-*N*-methylsalsolinol	12,800	2216

**Table 3:** The ΔGbinding energy (kcal/mol) and RMSD values (Å) of a molecular docking simulation result of five ligands and three standard compounds against DNMT1.

No	Compound Name	Virtual Screening		Rigid Docking		Flexible Docking	
		ΔGbinding	RMSD	ΔGbinding	RMSD	ΔGbinding	RMSD
1	C-7756	−12.89	1.047	−12.97	1.529	−14.45	1.563
2	C-5769	−10.11	1.210	−10.96	1.932	−13.17	1.560
3	C-1723	−10.23	1.723	−10.80	1.831	−12.51	1.691
4	C-2129	−9.71	1.594	−12.04	1.929	−12.43	1.495
5	C-2140	−10.32	0.979	−11.88	1.686	−12.42	1.685
S1	SAH	−9.19	1.322	−10.52	1.608	−11.02	1.563
S2	SAM	−5.64	2.347	−11.69	2.564	−12.05	2.372
S3	SFG	−8.33	1.396	−10.54	1.493	−10.67	1.492

**Table 4:** The molecular properties, druglikeness, and drug score predictions of five ligands and three standard compounds using DataWarrior software and OSIRIS Property Explorer online web service.

No	Compound Name	Molecular Weight (Da)	log P	H- Don	H- Acc	TPSA (Å^2^)	Rotatable Bond	Druglikeness	Drug Score
1	C-7756	492.78	1.97	2	3	24.67	10	1.45	0.41
2	C-5769	490.72	0.91	3	4	44.9	8	−2.25	0.23
3	C-1723	494.76	1.31	3	4	44.9	10	−1.11	0.34
4	C-2129	496.64	1.57	5	5	69.57	7	−0.61	0.33
5	C-2140	494.63	1.46	5	5	69.57	6	−0.89	0.24
S1	SAH	384.41	−3.72	4	11	212.38	7	−18.44	0.42
S2	SAM	399.45	−3.93	4	11	187.08	7	−9.09	0.39
S3	SFG	382.40	−3.95	5	12	214.72	7	−19.10	0.42

**Table 5:** The mutagenicity/carcinogenicity prediction of five ligands and three standard compounds using Toxtree v2.6.6 software.

No	Compound Name	Genotoxic Carcinogenicity	Non-Genotoxic Carcinogenicity	QSAR Carcinogenicity	Mutagenicity (Salmonella Typhimurium)
1	C-7756	No	No	No	No
2	C-5769	No	No	No	No
3	C-1723	No	No	No	No
4	C-2129	No	No	No	No
5	C-2140	No	No	No	No
S1	SAH	Yes	No	No	No
S2	SAM	Yes	No	No	No
S3	SFG	Yes	No	No	No

**Table 6:** The ADME-Tox prediction results of five ligands and three standard compounds using AdmetSAR software.

**No**	Compound Name	P-gp Substrate/Inhibi tor	CYP450 Substrate/Inhibi tor	CYP Inhibitory Promiscuity	Biodegradable	AMES Toxicity	Carcinogenicity
1	C-7756	Substrate, non-inhibitor	Substrate (CYP450 3A4), non-inhibitor	Low	Not Ready	None	None
2	C-5769	Substrate, non-inhibitor	Substrate (CYP450 3A4), non-inhibitor	Low	Not Ready	None	None
3	C-1723	Substrate, non-inhibitor	Substrate (CYP450 3A4), non-inhibitor	Low	Not Ready	None	None
4	C-2129	Substrate, non-inhibitor	Substrate (CYP450 3A4), non-inhibitor	Low	Not Ready	None	None
5	C-2140	Substrate, non-inhibitor	Substrate (CYP450 3A4), non-inhibitor	Low	Not Ready	None	None
S1	SAH	Substrate, non-inhibitor	Non-substrate, non-inhibitor	Low	Not Ready	None	None
S2	SAM	Substrate, non-inhibitor	Substrate (CYP450 3A4), non-inhibitor	Low	Not Ready	None	None
S3	SFG	Non-substrate, non-inhibitor	Non-substrate, non-inhibitor	Low	Not Ready	None	None

**Table 7:** The oral bioavailability, PAINS, and synthetic accessibility prediction results of five ligands and three standard compounds using SwissADME software.

No	Compound Name	GI Absorption	Lipinski	Veber	Egan	Bioavailability Score	PAINS	Synthetic Accessibility
1	C-7756	High	0	0	0	0.55	0	5.59
2	C-5769	High	0	0	0	0.55	0	6.09
3	C-1723	High	0	0	0	0.55	0	5.51
4	C-2129	High	0	0	0	0.55	1	5.11
5	C-2140	High	0	0	0	0.55	1	5.12
S1	SAH	Low	1	1	1	0.55	0	4.69
S2	SAM	Low	1	1	1	0.55	0	4.94
S3	SFG	Low	1	1	1	0.55	0	4.78

## Data Availability

The data presented in this research is available in this article.

## References

[1] Esteller M., Herman J.G. (2002). Cancer as an epigenetic disease: DNA methylation and chromatin alterations in human tumours. J. Pathol..

[2] Florin-Andrei Taran A., Schneeweiss A., Lux M.P., Janni W., Hartkopf A.D., Nabieva N., Overkamp F., Kolberg H.-C., Hadji P., Tesch H. (2018). Update Breast Cancer 2018 (Part 1)—Primary Breast Cancer and Biomarkers. Geburtshilfe Frauenheilkd..

[3] World Health Organisation Latest Global Cancer Data. https://www.who.int/cancer/PRGlobocanFinal.pdf.

[4] World Health Organisation Global Cancer Observatory (GLOBOCAN). https://gco.iarc.fr/.

[5] Lyko F. (2017). The DNA methyltransferase family: A versatile toolkit for epigenetic regulation. Nat. Rev. Genet..

[6] Gao F., Das S.K. (2014). Epigenetic regulations through DNA methylation and hydroxymethylation: Clues for early pregnancy in decidualization. Biomol. Concepts.

[7] Zhang Z., Liu S., Lin K., Luo Y., Perry J., Wang Y., Song J. (2015). Crystal Structure of Human DNA Methyltransferase 1. HHS Public Access.

[8] Moore L.D., Le T., Fan G. (2013). DNA methylation and its basic function. Neuropsychopharmacology.

[9] Lee E.Y.H.P., Muller W.J. (2010). Oncogenes and tumor suppressor genes. Cold Spring Harb. Perspect. Biol..

[10] Khandige S., Shanbhogue V.V., Chakrabarty S., Kapettu S. (2011). Methylation Markers: A Potential Force Driving Cancer Diagnostics Forward. Oncol. Res. Featur. Preclin. Clin. Cancer Ther..

[11] Lim W.J., Kim K.H., Kim J.Y., Jeong S., Kim N. (2019). Identification of DNA-methylated CpG islands associated with gene silencing in the adult body tissues of the ogye chicken using RNA-Seq and reduced representation bisulfite sequencing. Front. Genet..

[12] Janitz K., Janitz M. (2011). Assessing Epigenetic Information.

[13] Oberley M.J., Farnham P.J. (2003). Probing Chromatin Immunoprecipitates with CpG-Island Microarrays to Identify Genomic Sites Occupied by DNA-Binding Proteins. Methods Enzymol..

[14] Ropero S., Esteller M. (2005). 7 DNA methylation analysis of human cancer. Handbook of Immunohistochemistry and in Situ Hybridization of Human Carcinomas.

[15] el-Deiry W.S., Nelkin B.D., Celano P., Yen R.W., Falco J.P., Hamilton S.R., Baylin S.B. (1991). High expression of the DNA methyltransferase gene characterizes human neoplastic cells and progression stages of colon cancer. Proc. Natl. Acad. Sci. USA.

[16] Harvey A.L. (2008). Natural products in drug discovery. Drug Discov. Today.

[17] Bindseil K.U., Jakupovic J., Wolf D., Lavayre J., Leboul J., van der Pyl D. (2001). Pure compound libraries; a new perspective for natural product based drug discovery. Drug Discov. Today.

[18] Brueckner B., Garcia Boy R., Siedlecki P., Musch T., Kliem H.C., Zielenkiewicz P., Suhai S., Wiessler M., Lyko F. (2005). Epigenetic reactivation of tumor suppressor genes by a novel small-molecule inhibitor of human DNA methyltransferases. Cancer Res..

[19] Martinet N., Michel B.Y., Bertrand P., Benhida R. (2011). Small molecules DNA methyltransferases inhibitors. Medchemcomm.

[20] Kapetanovic I.M. (2008). Computer-aided drug discovery and development ( CADDD ): In silico -chemico-biological approach. Chem. Biol. Interact..

[21] de Ruyck J., Brysbaert G., Blossey R., Lensink M.F. (2016). Molecular docking as a popular tool in drug design, an in silico travel. Adv. Appl. Bioinforma. Chem..

[22] El-Saadi M.W., Williams-Hart T., Salvatore B.A., Mahdavian E. (2015). Use of in-silico assays to characterize the ADMET profile and identify potential therapeutic targets of fusarochromanone, a novel anti-cancer agent. Silico Pharmacol..

[23] Takeshita K., Suetake I., Yamashita E., Suga M., Narita H., Nakagawa A., Tajima S. (2011). Structural insight into maintenance methylation by mouse DNA methyltransferase 1 (Dnmt1). Proc. Natl. Acad. Sci. USA.

[24] Song J., Rechkoblit O., Bestor T.H., Patel D.J. (2011). Structure of DNMT1-DNA complex reveals a role for autoinhibition in maintenance DNA methylation. Science.

[25] Strausberg R.L., Feingold E.A., Grouse L.H., Derge J.G., Klausner R.D., Collins F.S., Wagner L., Shenmen C.M., Schuler G.D., Altschul S.F. (2002). Generation and initial analysis of more than 15,000 full-length human and mouse cDNA sequences. Proc. Natl. Acad. Sci. USA.

[26] Sterling T., Irwin J.J. (2015). ZINC 15—Ligand Discovery for Everyone. J. Chem. Inf. Model..

[27] Vilar S., Cozza G., Moro S. (2008). Medicinal chemistry and the molecular operating environment (MOE): Application of QSAR and molecular docking to drug discovery. Curr. Top. Med. Chem..

[28] Sander T., Freyss J., Von Korff M., Rufener C. (2015). DataWarrior: An open-source program for chemistry aware data visualization and analysis. J. Chem. Inf. Model..

[29] Sander T., Freyss J., Von Korff M., Reich J.R., Rufener C. (2009). OSIRIS, an entirely in-house developed drug discovery informatics system. J. Chem. Inf. Model..

[30] Contrera J.F. (2013). Validation of Toxtree and SciQSAR in silico predictive software using a publicly available benchmark mutagenicity database and their applicability for the qualification of impurities in pharmaceuticals. Regul. Toxicol. Pharmacol..

[31] Congreve M., Carr R., Murray C., Jhoti H. (2003). A “rule of three” for fragment-based lead discovery?. Drug Discov. Today.

[32] Benigni R., Bossa C., Jeliazkova N., Netzeva T., Worth A. (2008). The Benigni/Bossa Rulebase for Mutagenicity and Carcinogenicity—A Module of Toxtree.

[33] Cheng F., Li W., Zhou Y., Shen J., Wu Z., Liu G., Lee P.W., Tang Y. (2012). AdmetSAR: A comprehensive source and free tool for evaluating chemical ADMET properties. J. Chem. Inf. Model..

[34] Daina A., Michielin O., Zoete V. (2017). SwissADME: A free web tool to evaluate pharmacokinetics, drug-likeness and medicinal chemistry friendliness of small molecules. Sci. Rep..

[35] Liebeschuetz J.W., Cole J.C., Korb O. (2012). Pose prediction and virtual screening performance of GOLD scoring functions in a standardized test. J. Comput. Aided. Mol. Des..

[36] López-Camacho E., García-Godoy M.J., García-Nieto J., Nebro A.J., Aldana-Montes J.F. (2016). A new multi-objective approach for molecular docking based on rmsd and binding energy. Lecture Notes in Computer Science (Including Subseries Lecture Notes in Artificial Intelligence and Lecture Notes in Bioinformatics).

[37] Bell E.W., Zhang Y. (2019). DockRMSD: An open-source tool for atom mapping and RMSD calculation of symmetric molecules through graph isomorphism. J. Cheminform..

[38] Scoffin R., Slater M. (2015). The Virtual Elaboration of Fragment Ideas: Growing, Merging and Linking Fragments with Realistic Chemistry. Drug Discov. Dev. Deliv..

[39] Patlewicz G., Rodford R., Walker J.D. (2003). Quantitative structure-activity relationships for predicting mutagenicity and carcinogenicity. Environ. Toxicol. Chem..

[40] Xu C., Cheng F., Chen L., Du Z., Li W., Liu G., Lee P.W., Tang Y. (2012). In silico prediction of chemical Ames mutagenicity. J. Chem. Inf. Model..

[41] Finch A., Pillans P. (2014). P-glycoprotein and its role in drug-drug interactions. Australianprescriber.

[42] He J., Peng T., Yang X., Liu H. (2018). Development of QSAR models for predicting the binding affinity of endocrine disrupting chemicals to eight fish estrogen receptor. Ecotoxicol. Environ. Saf..

[43] Levine R.R. (1970). Factors affecting gastrointestinal absorption of drugs. Am. J. Dig. Dis..

[44] Egan W.J., Merz K.M., Baldwin J.J., Egan W.J., Kenneth M., Merz J., Baldwin J.J., Egan W.J., Merz K.M., Baldwin J.J. (2000). Prediction of Drug Absorption Using Multivariate Statistics. J. Med. Chem..

[45] Lipinski C.A. (2000). Drug-like properties and the causes of poor solubility and poor permeability. J. Pharmacol. Toxicol. Methods.

[46] Veber D.F., Johnson S.R., Cheng H., Smith B.R., Ward K.W., Kopple K.D. (2002). Molecular Properties That Influence the Oral Bioavailability of Drug Candidates. J. Med. Chem..

[47] Dahlin J.L., Nissink J.W.M., Strasser J.M., Francis S., Higgins L., Zhou H., Zhang Z., Walters M.A. (2015). PAINS in the assay: Chemical mechanisms of assay interference and promiscuous enzymatic inhibition observed during a sulfhydryl-scavenging HTS. J. Med. Chem..

[48] Fragomeni S.M., Sciallis A., Jeruss J.S. (2018). Molecular Subtypes and Local-Regional Control of Breast Cancer. Surg. Oncol. Clin. N. Am..

[49] Mitri Z., Constantine T., O’Regan R. (2012). The HER2 Receptor in Breast Cancer: Pathophysiology, Clinical Use, and New Advances in Therapy. Chemother. Res. Pract..

[50] Shin E., Lee Y.K., Koo J.S. (2016). Differential expression of the epigenetic methylation-related protein DNMT1 by breast cancer molecular subtype and stromal histology. J. Transl. Med..

[51] Saldívar-González F.I., Gómez-García A., Chávez-Ponce De León D.E., Sánchez-Cruz N., Ruiz-Rios J., Pilón-Jiménez B.A., Medina-Franco J.L. (2018). Inhibitors of DNA methyltransferases from natural sources: A computational perspective. Front. Pharmacol..

[52] Svedružić Ž.M. (2011). Dnmt1: Structure and Function.

[53] Kulis M., Esteller M. (2010). DNA Methylation and Cancer. Adv. Genet..

[54] Gnyszka A., Jastrzębski Z., Flis S. (2013). DNA Methyltransferase inhibitor and their emerging role in epigenetic therapu of cancer. Anticancer Res..

[55] Kumar A., Voet A., Zhang K.Y.J. (2012). Fragment Based Drug Design: From Experimental to Computational Approaches. Curr. Med. Chem..

[56] Lipinski C.A. (2004). Lead- and drug-like compounds: The rule-of-five revolution. Drug Discov. Today Technol..

[57] Giri S., Bader A. (2015). A low-cost, high-quality new drug discovery process using patient-derived induced pluripotent stem cells. Drug Discov. Today.

[58] Myers S., Baker A. (2001). Drug discovery—An operating model for a new era. Nat. Biotechnol..

[59] Noori H.R., Spanagel R. (2013). In silico pharmacology: Drug design and discovery’s gate to the future. Silico Pharmacol..

[60] Yang H., Sun L., Li W., Liu G., Tang Y. (2018). In Silico Prediction of Chemical Toxicity for Drug Design Using Machine Learning Methods and Structural Alerts. Front. Chem..

[61] Abad-Zapatero C. (2013). Chapter 5—Analysis of the Content of SAR Databases BT—Ligand Efficiency Indices for Drug Discovery. Expert Opinion on Drug Discovery.

[62] Lin J.H., Yamazaki M. (2003). Role of P-glycoprotein in pharmacokinetics: Clinical implications. Clin. Pharmacokinet..

[63] Moroy G., Martiny V.Y., Vayer P., Villoutreix B.O., Miteva M.A. (2012). Toward in silico structure-based ADMET prediction in drug discovery. Drug Discov. Today.

[64] Clarke S.E., Jones B.C. (2002). Human cytochromes P450 and their role in metabolism-based drug-drug interactions. Drug-Drug Interactions.

